# Maternal Pre-Pregnancy Obesity Is Associated with Altered Placental Transcriptome

**DOI:** 10.1371/journal.pone.0169223

**Published:** 2017-01-26

**Authors:** Signe Altmäe, Maria Teresa Segura, Francisco J. Esteban, Sabine Bartel, Pilar Brandi, Martin Irmler, Johannes Beckers, Hans Demmelmair, Carmen López-Sabater, Berthold Koletzko, Susanne Krauss-Etschmann, Cristina Campoy

**Affiliations:** 1 Department of Women’s and Children’s Health, Division of Obstetrics and Gynecology, Karolinska Institutet, Stockholm, Sweden; 2 Centre of Excellence for Paediatric Research EURISTIKOS and Department of Paediatrics, School of Medicine, University of Granada, Granada, Spain; 3 Department of Experimental Biology, University of Jaén, Jaén, Spain; 4 Division of Experimental Asthma Research, Research Center Borstel, Leibniz-Center for Medicine and Biosciences, Member of the German Center for Lung Research (DZL), Borstel, Germany; 5 Institute of Experimental Genetics, Helmholtz Zentrum Muenchen, Neuherberg, Germany; 6 Technische Universität München, Chair of Experimental Genetics, Freising, Germany; 7 German Center for Diabetes Research (DZD), Neuherberg, Germany; 8 Ludwig-Maximilians-University of Munich, Dr. Hauner Children’s Hospital, University of Munich Medical Centre, Munich, Germany; 9 Department of Nutrition and Bromatology, School of Pharmacy, University of Barcelona, Spain; 10 Institute for Experimental Medicine, Christian-Albrechts-Universitaet zu Kiel, Kiel, Germany; 11 Comprehensive Pneumology Center, Ludwig Maximilians University Hospital and Helmholtz Zentrum München, Großhadern, Germany; 12 Biohealth Institute of Granada, Granada, Spain; University of Southampton, UNITED KINGDOM

## Abstract

Maternal obesity has a major impact on pregnancy outcomes. There is growing evidence that maternal obesity has a negative influence on placental development and function, thereby adversely influencing offspring programming and health outcomes. However, the molecular mechanisms underlying these processes are poorly understood. We analysed ten term placenta’s whole transcriptomes in obese (n = 5) and normal weight women (n = 5), using the Affymetrix microarray platform. Analyses of expression data were carried out using non-parametric methods. Hierarchical clustering and principal component analysis showed a clear distinction in placental transcriptome between obese and normal weight women. We identified 72 differentially regulated genes, with most being down-regulated in obesity (n = 61). Functional analyses of the targets using DAVID and IPA confirm the dysregulation of previously identified processes and pathways in the placenta from obese women, including inflammation and immune responses, lipid metabolism, cancer pathways, and angiogenesis. In addition, we detected new molecular aspects of obesity-derived effects on the placenta, involving the glucocorticoid receptor signalling pathway and dysregulation of several genes including *CCL2*, *FSTL3*, *IGFBP1*, *MMP12*, *PRG2*, *PRL*, *QSOX1*, *SERPINE2* and *TAC3*. Our global gene expression profiling approach demonstrates that maternal obesity creates a unique *in utero* environment that impairs the placental transcriptome.

## Introduction

Maternal obesity has increased between 70–100% over the last decade [1]. This has had consequences for different aspects of female reproduction, as maternal adiposity is related to an increased risk of the majority of maternal and fetal complications [1]. Maternal pre-gravid obesity has been associated with an increased risk of miscarriage, gestational diabetes, pre-eclampsia, Caesarean section, instrumental vaginal delivery, birth trauma, stillbirth, and pelvic floor damage [1,2]. Infants of overweight and obese mothers are at higher risk of being born large for gestational age and/or macrosomic, and for developing obesity, cardiovascular disease, and diabetes in adulthood [2].

Being at the interface between the maternal and fetal environment, the placenta plays a central role in how maternal obesity influences the programming of health outcomes in the offspring. It has been shown that the placental structure and function is shaped by obesity already at the early developmental stages and onward [3]. Furthermore, previous studies demonstrate that obesity during pregnancy promotes a maternal environment favouring increased inflammation, lipotoxicity, and oxidative stress in the placenta [4–6], which may in turn alter maternal endothelial function [7], trophoblast invasion and differentiation [7], vascular development and function [6], and placental nutrient transport [8]. The molecular mechanisms underlying these changes are poorly understood so far and therefore further research in this field is warranted. Knowing the molecular bases of these processes would provide valuable insights into the placental development and functions and help to identify molecular mechanisms that have both immediate and long lasting effects on fetal health.

With the recent evolution of ‘omics’ techniques, a number of genome-scale transcriptional studies on normal human placenta have been performed and a unique placental transcriptome has been identified [9–14]. However, the effects of maternal obesity on the human placental transcriptome have received little attention, and only a few studies have been published [3,5,6]. Saben *et al*. performed RNA-sequencing on term placentas from lean and obese women and found dysregulation of genes related to lipid metabolism, angiogenesis, hormone activity and inflammation in placentas from obese women. This indicates that the obese maternal environment may adversely affect placental development and function [6]. Two other studies focused on the first trimester placental transcriptome using microarray platforms [3,5]. Lassance *et al*. analysed the transcriptome of trophoblast cells of first trimester placenta exposed *in vitro* to insulin and obesity. They concluded that maternal obesity associated with insulin resistance programs the placental transcriptome towards refractoriness to insulin with potential adverse effects for placental structure and function [3]. Another study by Saben *et al*. identified several genes and signalling pathways in trophoblast cells following lipotoxic challenge, providing novel insights into the possible mechanisms underlying obesity-induced placental inflammation [5]. Thus, there is only one study to date that has directly investigated how maternal obesity influences the placental transcriptome.

We therefore set out: (i) to investigate the transcriptome profile of term placentae in obese women in comparison to normal weight women in order to provide further insights into the molecular effects of maternal obesity on placental environment, and (ii) to find molecular biomarkers/targets and candidate regulatory pathways dysregulated by maternal pre-pregnancy obesity.

## Materials and Methods

### Study subjects

Ten women participating in the PREOBE study were carefully selected for the current study. The PREOBE study (Role of Nutrition and Maternal Genetics on the Programming of Development of Fetal Adipose Tissue) is an observational cohort study on healthy normal weight, overweight, and obese women, as well as women who developed gestational diabetes. This study was performed at the Clinical University Hospital ‘San Cecilio’ and ‘Mother-Infant’ Hospital in Granada, Spain (the study has been registered at www.ClinicalTrials.gov, NLM identifier: NCT01634464 2012) [15]. The project was approved by the Research Bioethical Committee of the University of Granada, and all women signed written informed consent after receiving the full information by a member of the research team.

Five normal weight women, whose pre-pregnancy body mass index (BMI kg/m^2^) was between 18–25, and five obese women, whose BMI was ≥30, were selected for this study. All women were: with European descent, healthy, with age range between 25 and 35 years, had no pregnancy complications or gestational diabetes, had normal blood pressure throughout pregnancy, were non-smoking, consumed no alcohol or drugs, had a normal pregnancy with fetal development in accordance to the pregnancy week, fetal position was cephalic, had natural term delivery, and had a normal/healthy placenta based on visual inspection. During pregnancy, routine pregnancy evaluation was performed at weeks 24, 34, and at delivery (see Table 1 for clinical characteristics). Additionally, blood was collected for fatty acid analysis during pregnancy and at delivery (the protocol for fatty acid analysis is described in our previous publication [16].

**Table 1:** Clinical characteristics of the participating women in the study and their perinatal outcomes.

	Normal weight (n = 5)	Obese (n = 5)	p-value
Maternal age (y)	30.2±1.9	29.8±4.6	NS
Parity (≥1)^a^	1 (20%)	4 (80%)	NS^b^
Height (m)	1.68±0.02	1.63±0.02	<0.01
Weight (pre-pregnancy)(kg)	59.6±5.7	89.3±6.4	<0.01
BMI (pre-pregnancy)(m/kg^2^)	21.0±2.0	33.6±2.0	<0.01
Weight at 24 weeks (kg)	66.3±6.0	91.5±4.3	<0.01
Weight at 34 weeks (kg)	71.2±7.8	93.8±5.4	0.02
Weight at delivery (kg)	72.0±9.8	97.5±11.0	0.03
BMI at week 24 (m/kg^2^)	23.4±2.1	34.4±1.4	<0.01
BMI at week 34 (m/kg^2^)	25.1±2.8	35.2±1.2	0.02
BMI at delivery (m/kg^2^)	25.2±3.3	36.7±3.6	0.03
Weight gain until week 34 (kg)	11.6±4.9	5.1±4.2	0.06
Total weight gain during pregnancy	12.5±6.7	6.8±6.9	NS
Gestational age at delivery (weeks)	38.8±0.8	39.7±1.3	NS
Placenta weight (g)	472±122	562±84	NS
Placental/fetal -ratio	0.14±0.04	0.16±0.03	NS
Apgar score at 5 min	10±0	10±0	NS
Infant gender (boy)^a^	3 (60%)	4 (80%)	NS^b^
Birth weight (g)	3344±302	3602±595	NS
Newborn birth length (cm)	49.6±1.5	51.2±2.7	NS
Newborn BMI (m/kg^2^)	13.6±0.9	13.7±1.4	NS
Newborn head circumference (cm)	35±0.7	35.2±2.1	NS
Newborn waist circumference (cm)	32.2±1.0	34.6±4.1	NS
Newborn waist/height index	0.64±0.03	0.68±0.07	NS

Data is presented as mean±SD or ^a^n (%), and p-values are non-parametric Mann-Whitney *U*-test or ^b^Chi square test. NS—statistically non-significant difference between groups. BMI—Body Mass Index

### Collection of term placenta samples

Placenta samples were collected and weighed immediately after delivery by a well-trained expert. Disc samples containing both maternal and fetal tissue were collected from identical portions of the placental plate starting from the periphery of the maternal side in order to avoid any regional variations. Next, after removal of the decidua, a 0.5 x 0.5 x 0.5 cm (200 mg) sample was excised from the middle of the radius (distance between the insertion of the umbilical cord and the periphery) of each placenta. Each sample was then rinsed twice with saline solution (NaCl 0.9%), and immediately placed into sterile 1.5 ml microtubes containing RNAlater solution (Quiagen, Venlo, The Netherlands). All samples were stored under RNase free conditions at -80°C for later analysis at the Comprehensive Pneumology Center Munich.

### Total RNA isolation and microarray hybridisation

For microarray and real-time PCR analysis, total RNA was isolated from the placental samples by using the miRNeasy mini kit (Quiagen) according to the manufacturer’s instructions. RNA concentration was calculated with a Nanodrop-1000 spectrophotometer (NanoDrop Fluorometer, Thermo Scientific, Wilmington, DE, USA). Only the highest quality RNA (260/280 ratio > 1.8, no degradation as detected by RNA agarose gel) was used for microarray analysis. 70% of the RNA samples were further analysed with Bioanalyzer (A2100 Bioanalyzer, Agilent Technologies). An RNA integrity value (RIN) of ≥7.0 was considered acceptable.

Thirty ng of total RNA was amplified using the Ovation PicoSL WTA System V2 in combination with the Encore Biotin Module (NuGEN Technologies, Inc, San Carlos, CA, USA). Amplified cDNA was hybridized on Affymetrix human Gene ST 2.0 arrays containing about 50,000 probe sets (Affymetrix, Santa Clara, CA, USA). Staining (Fluidics script FS450_00002) and scanning was done according to the Affymetrix expression protocol including minor modifications as suggested in the Encore Biotin protocol (NuGEN Technologies, Inc).

### Microarray data analysis

#### Pre-processing and differential gene expression

Expression console (v.1.2, Affymetrix) was used for quality control and to obtain annotated normalized RMA gene-level data (standard settings including median polish and sketch-quantile normalisation).

Data analyses were performed using the R-statistical software system (Free Software Foundation, Boston, USA). Gene expression profiles were determined by comparing the obese and normal weight groups (2 by 2 comparisons) by means of the rank product non-parametric test in the Bioconductor RankProd package (www.bioconductor.org/packages/devel/bioc/html/RankProd.html). Due to the limited number of samples, a non-parametric statistical test was used as a rough filter to narrow down the list of the most relevant genes. The statistics calculated is equivalent to the geometric mean rank and it is less sensitive to outliers. Additionally, the proportion of false positives (PFP) correction was used to control errors in multiple tests, as it effectively controls the accumulation of false-positives relative to the total number of positive results [17]. A PFP of <0.05 was considered statistically significant.

Our primary microarray data are available in the public database ArrayExpress (www.ebi.ac.uk/arrayexpress) under accession number E-MTAB-4541. Two samples did not pass the stringent Model Based Quality Control Assessment of Affymetrix GeneChips, which was carried out using the corresponding affyPLM package (https://bioconductor.org/packages/release/bioc/html/affyPLM.html). Briefly, the Normalized Unscaled Standard Errors (NUSE) function was run over the data. This process accounts for differences in variability between genes based on standard error (SE) estimates obtained for each gene on each array; any given array with elevated SE relative to the other arrays is considered of lower quality. The final analyses were performed in total on 8 samples (4 in each group).

#### Sample clustering and principal component analysis

In order to validate the above gene selection with a non-parametric method, a principal component analysis (PCA) and a hierarchical clustering were performed using MeV 4.2.02 software (www.tm4.org) [18]. In PCA analysis, a three-dimensional scatter plot was produced in order to visualize the differences between the sample sets based on each sample’s gene expression profile. The method used to calculate the distance was the covariance and the number of probes that differentially expressed genes. In the hierarchical clustering, the data were Z-normalized by gene and the Euclidean distance was selected as the similarity to cluster expression profiles. The method used to perform the linkage was a complete-linkage hierarchical clustering algorithm.

#### Enrichment analysis of the results

Enrichment analysis of differentially regulated genes was explored by using the Database for Annotation, Visualization and Integrated Discovery (DAVID, v. 6.7) [19] and gProfiler [20], and Ingenuity Pathways Analysis (IPA^®^) (Ingenuity^®^ Systems, www.ingenuity.com, release date 09.12.2014). DAVID searches blocks of functionally related genes according to different criteria such as the Gene Ontology (GO) terms as biological process, cellular component and molecular function. GO FAT search was used in order to filter the broadest terms so that they do not overshadow the more specific terms (david.abcc.ncifcrf.gov). Further enrichment analyses, including canonical pathways and networks of dysregulated genes were analysed with IPA. A multiple testing correction, false discovery rate (FDR) was applied, and FDR<5.0% and a p-value <0.05 were considered statistically significant.

### Microarray validation by real-time PCR

In order to validate the microarray results, 500ng of the same RNA samples were reverse-transcribed using the Quantitect Reverse Transcription kit according to the manufacturer’s recommendations (Qiagen, Venlo, Netherlands). The obtained cDNA was diluted 10x in PCR-grade water for qRT-PCR analysis.

The genes *AREG*, *CCL2*, *FSTL3*, *IGFBP1* and *MMP12* were used for microarray validation. Specific forward and reverse primer pairs were designed for each gene and are listed in Table A in S1 File. Real-time qPCR was performed on the Roche Light Cycler II platform with the Roche Light Cycler 480 SYBR Green I Master mix (Roche, Mannheim, Germany). The final products were verified by melting curve analysis by using the provided software (Roche, Mannheim, Germany). Data are presented as dCp values (Cp_targetgene_-Cp_referencegene_), where higher dCp values represent lower expression; while graphs are represented on reversed Y-axes. *B2M* and *YWHAZ* where used as reference genes. We tested 8 different housekeeping genes that have been used in studies of human placenta (by [21] and others) for our study: *SDHA*, *B2M*, *ACTB*, *BRD1*, *KCTD2*, *HPRT*, *YWHAZ* and *TBP*. *B2M* and *YWHAZ* genes were the most suitable for our study as they showed the lowest variation among subjects and groups. The geometric mean of *B2M* and *YWHAZ* Ct values was used for normalisation. The analysis of gene expression differences between the study groups was performed using the Mann-Whitney *U*-test. *P*-values of <0.05 were considered statistically significant.

## Results

### Characteristics of participating subjects

The perinatal outcomes between normal weight and obese women did not differ significantly. However, the mean weight of the placenta, as well as the newborn’s birth weight and length, head circumference, waist circumference, and BMI were somewhat bigger/higher in obese women than normal weight women (Table 1).

The analysis of plasma fatty acids at pregnancy weeks 24 and 34 demonstrated significant differences in palmitic acid, oleic acid and arachidonic acid concentrations between the study groups (fatty acid values are expressed as percentages by weight, wt %. See Table B in S1 File). At pregnancy weeks 24 and 34 palmitic acid was significantly higher in obese women when compared to normal weight women (33.7±0.6 *vs*. 30.5±1.0, p = 0.016; and 34.3±0.7 *vs*. 32.2±1.1, p = 0.032). Oleic acid was significantly lower (10.7±1.0 *vs*. 13.0±1.8, p = 0.032) and arachidonic acid was significantly higher (9.7±0.2 *vs*. 7.9±1.4, p = 0.016) in plasma of obese women when compared to normal weight women at pregnancy week 34.

### Cluster analysis of microarray data

**Fig 1:**
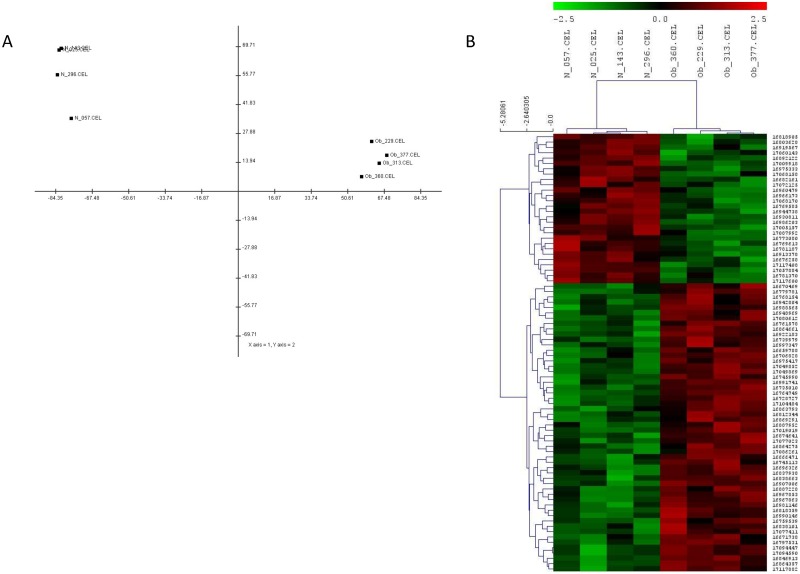
**(A)** Principal component analysis (PCA) of term placental gene expression profiles in obese (Ob_ N°) and normal weight women (N_N°). **(B)** Cluster analysis of dysregulated genes in term placentas from obese (Ob_ N°) *vs*. normal weight women (N_N°). Red represents genes with high expression levels and green represents genes with low expression levels.

The PCA analysis demonstrated a very clear separation of placental gene expression profiles between obese and normal weight women (Fig 1A). In addition, hierarchical clustering was applied to the microarray data and a similar pattern was obtained, where two study groups clustered into two different groups (Fig 1B).

### Differential gene expression analysis of term placenta from obese *vs*. normal weight women

A total of 72 differentially regulated genes were identified. Eleven transcripts were up-regulated and 61 genes were down-regulated in term placentas of obese *vs*. normal weight women (see Table 2). Interestingly, the majority of the differentially regulated genes were down-regulated (84.7%), while only 15.3% of the genes were up-regulated in the placentas of obese women.

**Table 2:** List of dysregulated genes in term placentas from obese women *vs*. normal weight women. The genes identified in previous transcriptome studies in human placentas in health and disease are indicated.

Gene symbol	Gene name	Biological process/function	FC	p-value (PFP)	Previous studies
*CCL2*	Chemokine ligand 2	Cytokine. Immune and inflammatory response	2.77	0.00	[9]
*RN5S363*	RNA, 5S ribosomal pseudogene 363	Unknown	2.72	0.02	
*RNY4P17*	RNA, Ro-associated Y4 pseudogene 17	Unknown	2.69	0.01	
*RN5S220*	RNA, 5S ribosomal pseudogene 220	Unknown	2.63	0.01	
*GRIK1-AS2*	GRIK1 antisense RNA 2	Transcription factor	2.43	0.01	
*HLA-DRB1*	Major histocompatibility complex, class II, DR beta 1	Immune response	2.32	0.04	[9]
*AREG*	Amphiregulin	Growth factor. Promotes growth of epithelial cells. Cancers and inflammatory conditions	2.15	0.02	
*DND1*	DND1 microRNA-mediated repression inhibitor 1	Inhibits microRNA-mediated repression. Cancer.	2.10	0.05	
*SNORA10*	Small nucleolar RNA, H/ACA box 10	Unknown	2.08	0.04	
*STARD10-AS1*	STARD10 antisense RNA 1	Unknown	2.01	0.04	
*RNF144B*	Ring finger protein 144B	Apoptosis	1.88	0.04	[9]
*ERAP2*	Endoplasmic reticulum aminopeptidase 2	Immune response	-1.91	0.04	[22]
*C12orf75*	Chromosome 12 open reading frame 75	Unknown	-1.91	0.03	[23]
*MGAT5*	Mannosyl-glycoprotein beta-1,6-N-acetyl-glucosaminyltransferase	Enzyme. Biosynthesis of glycoprotein oligosaccharides	-1.93	0.04	
*LGALS3*	Lectin, galactoside-binding, soluble, 3	Immune response, apoptosis, cell adhesion	-1.94	0.03	[9,12]
*PIPOX*	Pipecolic acid oxidase	L-lysine catabolic process, tetrahydrofolate metabolic process	-1.95	0.04	[9]
*ADAM19*	ADAM metallopeptidase domain 19	Cell migration and adhesion. Cancer, inflammatory diseases	-1.97	0.04	
*CERS6*	Ceramide synthase 6	Ceramide biosynthetic process, shpingolipid metabolic process	-1.99	0.05	
*DDX46*	DEAD (Asp-Glu-Ala-Asp) box polypeptide 46	Pre-mRNA splicing	-1.99	0.03	
*TIMP3*	TIMP metallopeptidase inhibitor 3	Negative regulation of endopeptidase activity	-2.03	0.03	[9,10]
*FSTL3*	Follistatin-like 3	Cell differentiation, development	-2.03	0.03	[9,14,24–27]
*PDE10A*	Phosphodiesterase 10A	Signal transduction, blood coagulation	-2.04	0.02	
*RBP4*	Retinol binding protein 4, plasma	Retinol carrier in the blood. Developmental processes	-2.07	0.04	[25]
*ABI3BP*	ABI family, member 3 binding protein	Collagen and heparin binding	-2.08	0.02	[12]
*LOC100508885*	Uncharacterised LOC100508885	Unknown	-2.11	0.01	
*CRABP2*	Cellular retinoic acid binding protein 2	Retinoid signalling pathway. Development	-2.12	0.02	[9]
*GPRC5A*	G protein-coupled receptor, class C, group 5, member A	Retinoid acid and G protein signalling pathways. Development, growth and differentiation processes	-2.12	0.01	
*AOC1*	Amine oxidase, copper containing 1	Metal-binding membrane glycoprotein that oxidatively deaminates putrescine, histamine	-2.13	0.01	
*CCDC144A*	Coiled-coil domain containing 144A	Unknown	-2.17	0.03	[9]
*PLAC8*	Placenta-specific 8	Defence response	-2.17	0.01	[9,10,12]
*TSIX*	TSIX transcript, XIST antisense RNA	Unknown	-2.17	0.00	
*QSOX1*	quiescin Q6 sulfhydryl oxidase 1	Growth regulation	-2.20	0.01	[9,14]
*HN1*	Haematological and neurological expressed 1	Cancer	-2.21	0.01	[9,12,25]
*REPS2*	RALBP1 associated Eps domain containing 2	Inhibits growth factor signalling, cancer	-2.21	0.01	
*CGB8*	Chorionic gonadotropin, beta polypeptide 8	Produced in placenta and stimulates steroid synthesis in ovaries	-2.23	0.01	[9]
*MIR374B*	MicroRNA 374b	Gene expression regulation	-2.24	0.05	
*ADAM28*	ADAM metallopeptidase domain 28	Cell-cell and cell-matrix interactions, fertilization, muscle development, neurogenesis	-2.24	0.01	[9]
*TNFSF10*	Tumor necrosis factor superfamily, member 10	Cytokine, induces apoptosis	-2.25	0.01	[5,9,11,25]
*SERPINE2*	Serpin peptidase inhibitor, clade E, member 2	Negative regulation of blood coagulation, cell growth	-2.27	0.01	[10,14]
*GLIPR1*	GLI pathogenesis-related 1	Cellular lipid metabolic process, cancer	-2.30	0.01	[9,12]
*IL2RB*	Interleukin 2 receptor, beta	IL2 binding, immune response	-2.31	0.01	[9,10,12]
*SMYD3-IT1*	SMYD3 intronic transcript 1	Unknown	-2.32	0.01	
*UPK1B*	Uroplakin 1B	Cell development, activation, growth and motility	-2.35	0.02	[23]
*FOSB*	FBJ murine osteosarcoma viral oncogene homolog B	Regulator of cell proliferation, differentiation, transformation	-2.38	0.01	[13,24]
*OMD*	Osteomodulin	Carbohydrate metabolism, cell adhesion	-2.38	0.01	
*UBL3*	Ubiquitin-like 3	Unknown	-2.40	0.00	
*UGDH*	UDP-glucose 6-dehydrogenase	Biosynthesis of glycosaminoglycans. Signal transduction, cell migration, cancer growth	-2.43	0.01	[9,12]
*HSD11B1*	Hydroxysteroid dehydrogenase 1	Enzyme catalysing cortisol to cortisone. Obesity, insulin resistance	-2.47	0.01	[9,25]
*SLPI*	secretory leukocyte peptidase inhibitor	Immune response	-2.48	0.01	[9]
*NOTCH2NL*	Notch 2 N-terminal like	Notch signalling pathway, cell differentiation	-2.51	0.02	[9]
*DNAPTP3*	Histone demethylase UTY-like	Unknown	-2.52	0.00	
*FN1*	Fibronectin 1	Cell adhesion and migration, embryogenesis, wound healing, host defence, cancer	-2.55	0.00	[23]
*IL1R2*	Interleukin 1 receptor, type II	Immune response	-2.56	0.00	[9,12,25]
*EPYC*	Epiphycan	Fibrillogenesis, pregnancy	-2.60	0.01	[25]
*PAEP*	Progestagen-associated endometrial protein	Regulates uterine environment for pregnancy, organismal development	-2.65	0.01	[9]
*GKN1*	Gastrokine 1	Positive regulation of cell division and proliferation. Cancer	-2.68	0.00	
*RN5S457*	RNA, 5S ribosomal pseudogene 457	Unknown	-2.74	0.01	
*LOC728643*	Heterogeneous nuclear ribonucleoprotein A1 pseudogene		-2.79	0.00	
*HTRA4*	HtrA serine peptidas 4	Cell growth regulation	-2.90	0.00	[9,25]
*NOTUM*	Notum pectinacetylesterase homolog	Unknown	-3.01	0.00	[9]
*PLA2G7*	Phospholipase A2, group VII	Lipid catabolic process, positive regulation of inflammatory response	-3.12	0.00	[9,12]
*RN5S395*	RNA, 5S ribosomal pseudogene 395	Unknown	-3.16	0.00	
*DKK1*	Dickkopf WNT signalling pathway inhibitor 1	Embryonic development through inhibiting WNT signalling pathway	-3.46	0.00	[6,9,12]
*XIST*	X inactive specific transcript	X chromosome inactivation	-3.56	0.00	[10]
*LAIR2*	Leukocyte-associated immunoglobulin-like receptor 2	Immune response, inhibition of platelet aggregation and vessel formation during placental implantation	-3.95	0.00	[9]
*PRL*	Prolactin	Hormone, growth factor, immune response, supresses apoptosis, essential for lactation	-3.98	0.00	[24]
*MMP12*	Matrix metallopeptidase 12	Embryonic development, reproduction, tissue remodelling, inflammation, cancer	-4.00	0.00	[9,27]
*SNORD14E*	Small nucleolar RNA, C/D box 14E	Unknown	-4.22	0.00	
*CHRDL1*	Chordin-like 1	Eye development, BMP signalling pathway	-4.37	0.00	
*TAC3*	Tachykinin 3	Neuropeptide signalling pathway, pregnancy-related hypertension and pre-eclampsia	-5.04	0.00	[28]
*PRG2*	Proteoglycan 2, bone marrow	High levels in placenta, defence mechanisms and immune response	-6.55	0.00	[9]
*IGFBP1*	Insulin-like growth factor binding protein 1	Insulin receptor signalling pathway, positive regulation of cell growth, tissue regeneration	-10.27	0.00	[12,24–27]

GO analysis of differentially regulated genes revealed that a significant proportion of the genes in the placentae of obese women *vs*. normal weight women were involved in carbohydrate binding (11.5%, p = 0.009), and more specifically in polysaccharide (9.8%, p = 0.001) and heparin binding (8.2%, p = 0.003). A large proportion of the differentially regulated genes were located in the extracellular region (49.2%, p<0.0001).

**Fig 2:**
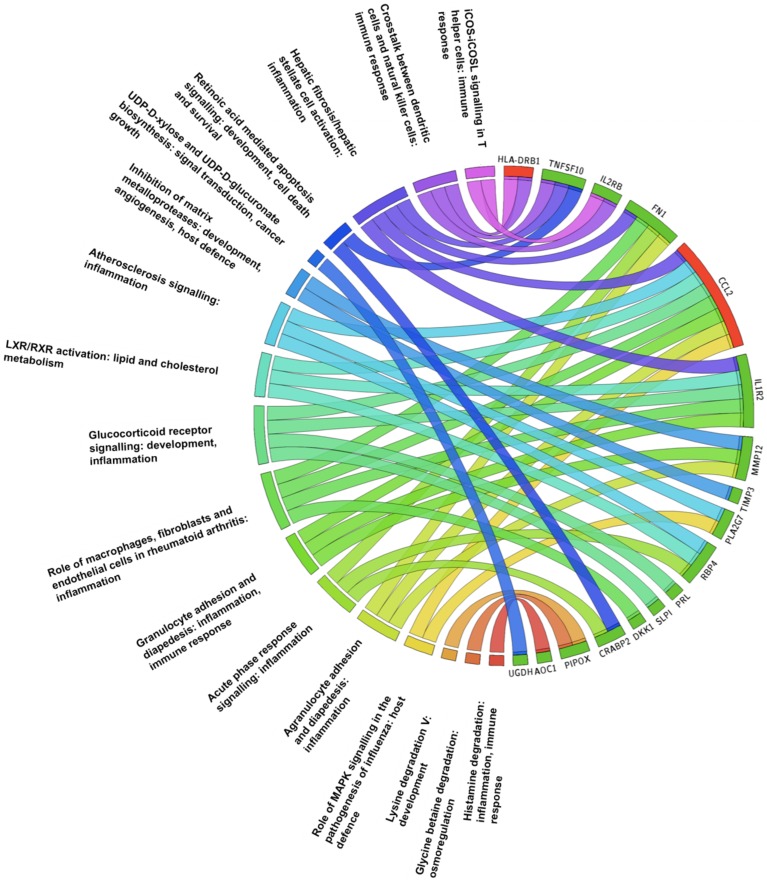
The Circos plot represents significantly enriched pathways associated with regulated genes in the placentae of obese women *vs*. normal weight women, detected using the Ingenuity Pathway Analysis library of canonical pathways. Outside the circle dysregulated genes and IPA pathways together with biological process are indicated. All genes are down-regulated (highlighted in green), except for HLA-DRB1 and CCL2 genes that are up-regulated (highlighted in red).

To obtain insight into relevant biological processes we used IPA analysis, and found that differentially regulated placental gene expression in obese women involved pathways concerning crosstalk between dendritic cells and natural killer cells, hepatic fibrosis/hepatic stellate cell activation, UPD-D-xylose and UPD-D-glucuronate biosynthesis, inhibition of matrix metalloproteases, atherosclerosis signalling among several others (Fig 2).

**Fig 3:**
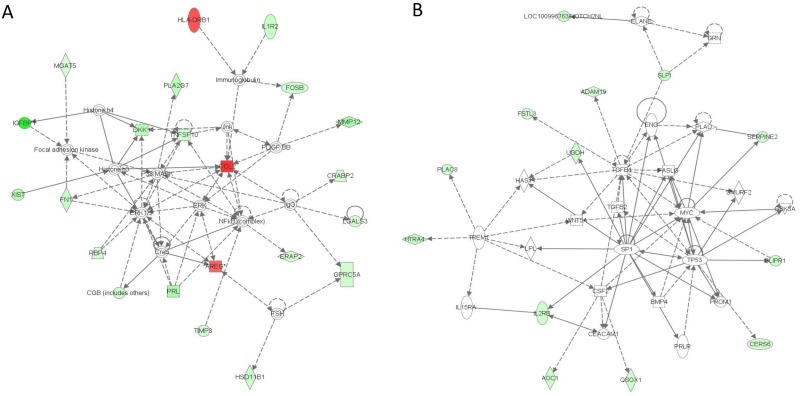
Shows the two highest scoring networks in IPA derived from the analysis of dysregulated genes in placentas of obese women *vs*. normal weight women. (**A)** Genes involved in embryonic development, organismal development and cancer. (**B)** Dysregulated genes involved in cellular movement, haematological system development and function, and immune cell trafficking. The intensity of the node colour indicates the degree of up- (red) or down- (green) regulation of gene expression. A white node represents a gene that is not part of our dataset, but is incorporated into the network through relationships with other genes. Nodes are displayed using various shapes that represent the functional class of the gene product, and a biological relationship between two nodes is represented as a line. Detailed information about the figure symbols can be found at www.ingenuity.com.

Analysis of the molecular relationships between differentially expressed genes showed two complex networks where the majority of signals were mediated through ERK, IgG, NFKB, and MAPK complexes (Fig 3A), and through TGFB, MYC and TP53 complexes (Fig 3B). The highest scoring IPA network of the molecular relationships between differentially expressed genes revealed the involvement of the molecules in top diseases and functions such as embryonic development, organismal development, and cancer, where different genes including: *AREG*, *CCL2*, *FN1*, *HLA-DRB1*, *IGFBP1*, *IL1R2*, *MMP12*, *PRL*, and *TNFSF10* were intertwining (IPA score of 46; Fig 3A). The second largest network of genes were involved in cellular movement, haematological system development and function, as well as immune cell trafficking, where different genes such as those for *ADAM19*, *AOC1*, *IL2RB*, *PLAC8*, *SERPINE2*, *SP1* and others seem to play important roles (IPA score of 23; Fig 3B).

The IPA Upstream Regulator Analysis identified two potential upstream regulators with a significant activation score (z-score ≥|2|) among the dysregulated genes in placentas of obese *vs*. normal weight women. IgG was predicted to be an activated upstream regulator (z-score = 2.0, p = 0.0004), with *CCL2*, *CRABP2*, *GPRC5A*, *LGALS3* as known target molecules. The second potential upstream regulator was ERBB2, being inhibited (z-score = -2.0, p = 0.002), with genes *ADAM19*, *CGB8*, *FN1*, and *FSTL3* as downstream targets.

### Microarray validation

**Fig 4:**
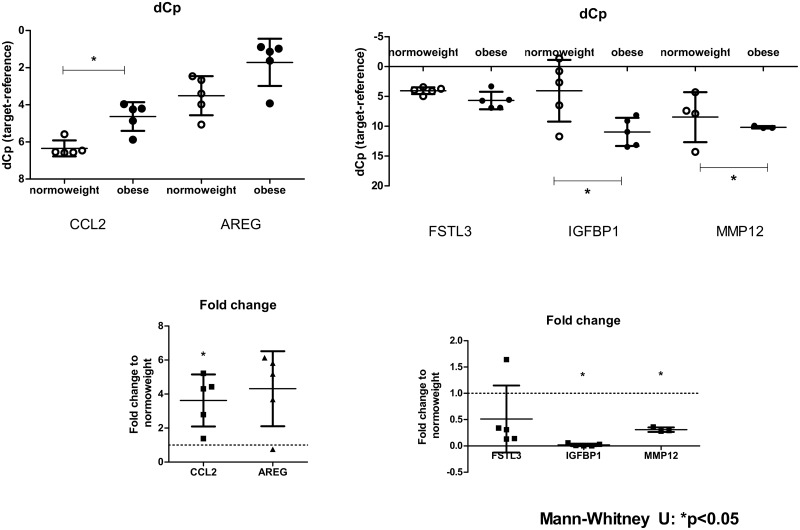
Microarray validation by real-time PCR. The expression levels of genes *AREG*, *CCL2*, *FSTL3*, *IGFBP1* and *MMP12* from our gene array analysis in comparison with real-time PCR gene expression levels are presented. QRT-PCR data are shown as dCp values (Cp_targetgene_-Cp_referencegene_), where higher dCp values represent lower expression and therefore, graphs are represented with reversed Y-axis. Mann-Whitney *U*-test *p <0.05, n = 5 samples/group.

Real-time PCRs using genes *AREG*, *CCL2*, *FSTL3*, *IGFBP1* and *MMP12* confirmed the array results. *CCL2* was significantly up-regulated, and *IGFBP1* and *MMP12* were significantly down-regulated (Fig 4). *AREG* and *FSTL3* genes expression profiles between obese and normal weight women did not reach statistical significance (p>0.05), nevertheless we observed a trend towards a similar dysregulation compared to the microarray.

## Discussion

We report the genome-wide transcriptome analysis of the effects of maternal obesity on the term placenta, in the absence of gestational diabetes and other complications. Our study results demonstrate that maternal pre-pregnancy obesity has adverse effects on the placental transcriptome, where previously characterised molecules and molecular pathways involved in placental development and function [14] were dysregulated. In addition, several new dysregulated molecules and signalling pathways were identified.

It is now well established that maternal obesity is associated with a pro-inflammatory milieu during pregnancy and in the placenta, [5,6]. In line with that, obese women in the present study had significantly higher plasma levels of arachidonic acid (which is involved in inflammatory processes) at pregnancy week 34. It has been demonstrated in mice that obesity during pregnancy disrupts inflammation through increased macrophage activation and elevation of cytokine gene expression, which provides potential links between placental inflammation and the programming of offspring disease by maternal obesity [29]. Furthermore, the majority of the identified dysregulated genes in our study including *CCL2*, *HLA-DRB1*, *IL1R2*, *IL2RB*, *TNFSF10*, *FN1*, and *MMP12* are involved in inflammation and immune responses, and the second biggest network of the dysregulated genes being involved in immune cell trafficking. We also identified immunoglobulin G (IgG) as an upstream activator of several dysregulated genes (*CCL2*, *CRABP2*, *GPRC5A*, *LGALS3*) in placentas from obese women. IgG is the main type of antibody found in blood and extracellular fluid that controls infection in body tissue by mediating pro- and anti-inflammatory activities [30].

Lipid metabolism is another process that has shown to be altered in placentas from obese women [6,7]. Our obese women demonstrated different palmitic acid and oleic acid levels in the plasma at pregnancy weeks 24 and 34 when compared to normal weight women, supporting altered lipid metabolism. Furthermore, functional analyses of our microarray data identified lipid and cholesterol metabolism, specifically the LXR/RXR activation pathway to be dysregulated among placentas from obese women. The importance of liver X receptors (LXRs) in physiological lipid and cholesterol metabolism suggests that they are involved in the development of metabolic disorders such as hyperlipidaemia (lipotoxicity) and atherosclerosis [31]. Indeed, a recent study concluded that maternal obesity leads to a lipotoxic placental environment that is associated with decreased regulators of angiogenesis and increased markers of inflammation and oxidative stress [6].

Another interesting finding in our study was that all analyses indicated that obesity influences cancer pathways. The detection of cancer pathways among placental gene expression is not surprising, as many proliferative, invasive, and immune tolerance mechanisms that support normal pregnancy are also exploited by malignancies [12,32]. Furthermore, we identified erb-b2 receptor tyrosine kinase 2 (ERBB2) as an upstream inhibitor of several genes including *ADAM19*, *CGB8*, *FN1*, and *FSTL3*. ERBB2 is involved in the mitogen-activated protein kinase signalling pathway and its role in cancer development and evolution has been shown [33].

In line with the similarities in pregnancy and cancer pathways, we detected haematological system development and function in network analysis, and angiogenesis as important biological processes affected by the dysregulated genes in placentas from obese women. Angiogenesis is a crucial process for fetomaternal exchanges and placental development, and alterations in this are associated with different pregnancy-related pathologies [34]. This is in accordance with a previous study that has detected altered regulation of genes related to angiogenesis in placentas from obese women [6].

A new observation in our study was the dysregulation of glucocorticoid receptor signalling pathway in placentas from obese women, where expression of genes *CCL2*, *IL1R2*, *PRL*, and *SLPI* were altered. Obesity has been associated with reduced sensitivity to glucocorticoid feedback, an effect believed to be mediated via altered sensitivity to the glucocorticoid receptor [35]. Glucocorticoid receptor signalling is regulating genes controlling the development, metabolism and immune responses, and is also involved in major organ systems physiology and pathophysiology in the human body. Its important role during gestation for postnatal survival as well as during embryonic development has been shown in different animal studies [35].

Another important finding in the search of obesity-related effects on term placentas was the identification of several potential target molecules that could have important roles in understanding how the placenta affects fetal development and obesity-derived offspring’s future health problems. We detected dysregulation in several genes that have been shown to play a role in the function of normal placental development in humans, including *AREG* [36], *CCL2* [37], *FOSB* [38], *GKN1* [39], *SERPINE2* [40], *SLPI* [41], and *XIST* [42]. In addition, several of the identified dysregulated genes, including *CCL2*, *CGB8*, *FOSB*, *FSTL3*, *HSD11B*, *IGFBP1*, *PRL*, *RBP4*, and *TAC3* have been implicated in adverse pregnancy outcomes such as intrauterine growth restriction [23,25,26,28], large for gestational age [24], and recurrent miscarriage [43]. Furthermore, a set of the dysregulated genes has been associated with the risk of developing pre-eclampsia, including *c12orf75* [23], *DKK1* [44], *FSTL3* [27], *HTRA4* [45], *IGFBP1* [27], *LAIR2* [27], *MMP12* [27], *PAEP* [27], and *UPK1B* [23]. Especially interesting and promising target molecules for obesity-derived implications in placenta could be *CCL2*, *PRL*, *MMP12*, *TAC3*, *PRG2* and *IGFBP1* that were the most dysregulated genes among our study group. Additionally, genes *FSTL3*, *QSOX1* and *SERPINE2* could serve as novel obesity-related biomarkers, as they have been shown to be uniquely enriched in the placenta [14], and we detected them as down-regulated among the placentas from obese women. All of these genes could serve as molecular biomarkers for potential progression towards metabolic syndrome in children that were born to overweight mothers.

A potential weakness of our and similar studies [5,10,11,22] is the small sample size. Nonetheless, cluster analyses showed a very clear separation between the groups in the present study. In addition, we have applied stringent non-parametric data analysis. Our study was strengthened by the well selected and characterised homogenous study groups, where healthy, young, non-smoking, Spanish women with no pregnancy complications and with natural delivery were enrolled.

## Conclusions

The placental metabolic abnormalities resulting from the effects of maternal obesity (e.g. lipotoxicity) may be transmitted to the offspring via *in utero* programming and hence there could be far-reaching consequences for offspring health. Management of maternal body weight and/or manipulation of lipid metabolism using both lifestyle and pharmacological interventions may provide benefits to the obese women as well as to their offspring. In fact, it has been shown that maternal weight loss by pre-pregnancy bariatric surgery prevented transmission of obesity to offspring compared with children of obese mothers who did not undergo this surgery [46]. Further improvements/intervention programs in maternal weight control (preferably before pregnancy [47]) would improve perinatal metabolic outcomes.

Our findings provide a fundamental resource for better understanding the complex effects of maternal pre-pregnancy obesity on placental transcriptome. We have detected new molecular aspects of obesity-derived effects on placentas, where the glucocorticoid receptor signalling pathway and dysregulation of several genes including *CCL2*, *FSTL3*, *IGFBP1*, *MMP12*, *PRG2*, *PRL*, *QSOX1*, *SERPINE2* and *TAC3* might have important roles. Our study also confirms the dysregulation of previously identified important processes and pathways among placentas from obese women. This global gene expression profiling approach demonstrates and confirms that maternal obesity creates a unique *in utero* environment that impairs the placental transcriptome. Further elucidation of normal and aberrant placental ‘ome’ regulation will not only increase our understanding of the origins of a disease, but may also provide novel approaches for intervention.

## Supporting Information

S1 File **Table A.** Real-time PCR primer sequences used for microarray validation. **Table B.** Fatty acids in plasma of participating women throughout the pregnancy.
(DOCX)
