# Supplementary material for: Maternal Pre-Pregnancy Obesity Is Associated with Altered Placental Transcriptome
Source: PLoS One. 2017 Jan 26;12(1):e0169223. doi: 10.1371/journal.pone.0169223 (PMC5268451; doi:10.1371/journal.pone.0169223)
Supplement: S1 File — Table A. Real-time PCR primer sequences used for microarray validation. Table B. Fatty acids in plasma of participating women throughout the pregnancy. (DOCX) [file pone.0169223.s001.docx]

**Supplementary Material**

**Table A.** Real-time PCR primer sequences used for microarray validation.

| **Gene** | **NCBI Ref Sequence** | **Forward (5'-3')** | **Reverse (5'-3')** |
| --- | --- | --- | --- |
| CCL2 | NM_002982 | GATCTCAGTGCAGAGGCTCG | TTTGCTTGTCCAGGTGGTCC |
| AREG | NM_001657 | CGCTCTTGATACTCGGCTCA | CCCCAGAAAATGGTTCACGC |
| IGFBP | NM_000596 | GGCTCTCCATGTCACCAACA | CCATTCCAAGGGTAGACGCA |
| FSTL3 | NM_005860 | ACACTCCTTAGAGCCCGGAT | AGGTCTTAGGGTAGGGGCTG |
| MMP12 | NM_002426 | GCTGTCACTACCGTGGGAAA | GGCAAGGTTGGCCATAAGGA |
| YWHAZ | NM_001135699.1 | ACTGGGTCTGGCCCTTAAC | GCGTGCTGTCTTTGTATGAC |
| B2M | NM_004048.2 | AGTATGCCTGCCGTGTGAAC | TGCGGCATCTTCAAACCTCC |

**Table B.** Fatty acids in plasma of participating women throughout the pregnancy.

| Fatty acids | Normal weight (n=5) | Obese (n=5) | p-value |
| --- | --- | --- | --- |
| Week 24 of pregnancy | | | |
| Myristic acid (C14:0) | 0.3±0.1 | 0.3±0.1 | NS |
| Palmitic acid (C16:0) | 30.5±1.0 | 33.7±0.6 | 0.016 |
| Oleic acid (C18:1) | 11.4±1.3 | 10.9±0.9 | NS |
| Linoleic acid (C18:2) | 25.6±2.2 | 24.0±1.0 | NS |
| Arachidonic acid (AA)(C20:4) | 8.9±1.7 | 9.5±0.5 | NS |
| Eicosapentaenoic acid (EPA)(C20:5) | 0.6±0.9 | 0.2±0.1 | NS |
| Docosahexaenoic acid (DHA)(22.6) | 4.5±1.9 | 3.6±0.7 | NS |
| Week 34 of pregnancy | | | |
| Myristic acid (C14:0) | 0.3±0.1 | 0.3±0.1 | NS |
| Palmitic acid (C16:0) | 32.2±1.1 | 34.3±0.7 | 0.032 |
| Oleic acid (C18:1) | 13.0±1.8 | 10.7±1.0 | 0.032 |
| Linoleic acid (C18:2) | 25.0±1.2 | 24.9±0.9 | NS |
| Arachidonic acid (AA)(C20:4) | 7.9±1.4 | 9.7±0.2 | 0.016 |
| Eicosapentaenoic acid (EPA)(C20:5) | 0.2±0.1 | 0.2±0.0 | NS |
| Docosahexaenoic acid (DHA)(22.6) | 3.8±1.0 | 3.4±0.2 | NS |
| Delivery | | | |
| Myristic acid (C14:0) | 0.3±0.0 | 0.3±0.1 | NS |
| Palmitic acid (C16:0) | 32.9±1.5 | 34.6±1.1 | NS |
| Oleic acid (C18:1) | 12.1±0.9 | 11.6±0.8 | NS |
| Linoleic acid (C18:2) | 25.6±2.2 | 23.2±1.5 | NS |
| Arachidonic acid (AA)(C20:4) | 8.2±1.3 | 9.7±1.5 | NS |
| Eicosapentaenoic acid (EPA)(C20:5) | 0.2±0.1 | 0.2±0.1 | NS |
| Docosahexaenoic acid (DHA)(22.6) | 3.7±1.1 | 3.5±0.6 | NS |

Fatty acid values are expressed as percentages by weight (wt %) of total detected fatty acids. Results are presented as mean±SD (standard deviation). Non-parametric Mann-Whitney *U*-test was used for comparing groups. NS – statistically non-significant difference between groups.
